# Mapping Multi-factor-mediated Chromatin Interactions to Assess Dysregulation of Lung Cancer-related Genes

**DOI:** 10.1016/j.gpb.2023.01.004

**Published:** 2023-01-23

**Authors:** Yan Zhang, Jingwen Zhang, Wei Zhang, Mohan Wang, Shuangqi Wang, Yao Xu, Lun Zhao, Xingwang Li, Guoliang Li

**Affiliations:** 1 National Key Laboratory of Crop Genetic Improvement, Huazhong Agricultural University, Wuhan 430070, China; 2 Hubei Key Laboratory of Agricultural Bioinformatics and Hubei Engineering Technology Research Center of Agricultural Big Data, 3D Genomics Research Center, Huazhong Agricultural University, Wuhan 430070, China

**Keywords:** Lung cancer, 3D genome, ChIA-PET, Chromatin interaction, Dysregulation

## Abstract

Studies on the **lung cancer** genome are indispensable for developing a cure for lung cancer. Whole-genome resequencing, genome-wide association studies, and transcriptome sequencing have greatly improved our understanding of the cancer genome. However, **dysregulation** of long-range **chromatin interactions** in lung cancer remains poorly described. To better understand the three-dimensional (3D) genomic interaction features of the lung cancer genome, we used the A549 cell line as a model system and generated high-resolution chromatin interactions associated with RNA polymerase II (RNAPII), CCCTC-binding factor (CTCF), enhancer of zeste homolog 2 (EZH2), and histone 3 lysine 27 trimethylation (H3K27me3) using long-read chromatin interaction analysis by paired-end tag sequencing (**ChIA-PET**). Analysis showed that EZH2/H3K27me3-mediated interactions further repressed target genes, either through loops or domains, and their distributions along the genome were distinct from and complementary to those associated with RNAPII. Cancer-related genes were highly enriched with chromatin interactions, and chromatin interactions specific to the A549 cell line were associated with oncogenes and tumor suppressor genes, such as additional repressive interactions on *FOXO4* and promoter–promoter interactions between *NF1* and *RNF135*. Knockout of an anchor associated with chromatin interactions reversed the dysregulation of cancer-related genes, suggesting that chromatin interactions are essential for proper expression of lung cancer-related genes. These findings demonstrate the 3D landscape and gene regulatory relationships of the lung cancer genome.

## Introduction

Lung cancer is currently one of the most lethal and common cancers [1]. A series of high-throughput sequencing studies have identified critical point mutations, structural variations, and copy number variations associated with tumorigenesis in lung cancer [2], [3], [4], [5], greatly augmenting our understanding of the cancer genome. However, many risk-related single nucleotide polymorphisms (SNPs) and genomic variations are found in regulatory and intergenic regions [6]. Linking these distal regulatory elements to their target genes is crucial for a more comprehensive understanding of the cancer genome and the potential functions of non-coding regions.

Chromosome conformation capture technologies, such as chromatin interaction analysis by paired-end tag sequencing (ChIA-PET) [7], can be used to explore the three-dimensional (3D) genome structure and link distal regulatory elements to their target genes. Many diseases are associated with dysregulation of the spatial structure of the genome. For example, disruption of topologically associated domains (TADs) can lead to abnormal development and malformation by preventing interactions between *Shh* gene and its limb enhancer [8]. Previous studies have used long-range interactions to map non-coding SNPs to target genes in patients with schizophrenia [9]. The 3D architecture of the genome is altered in many cancers [10], [11], [12], [13]. Recent research used high-throughput chromosome conformation capture (Hi-C) to characterize the larger-scale 3D genomic structures in lung cancer [14]. Therefore, clarifying 3D genomic disorders in cancer cells may be an innovative way to conduct cancer research. However, 3D information related to gene expression regulation in lung cancer requires further study.

Polycomb repressive complex 2 (PRC2) consists of core subunits, *i.e.*, enhancer of zeste homolog 2 (EZH2), suppressor of zest 12 (SUZ12), and embryonic ectoderm development (EED), and is critical for normal development and silencing of remote genes [15], [16], [17], [18], [19]. PRC2-associated chromatin interactions play an irreplaceable role in gene silencing [20], [21]. Studies have also shown that histone 3 lysine 27 trimethylation (H3K27me3)-enriched domains have distinct intradomain interactions that play essential roles during development and in cancer cells [22], [23]. Thus, EZH2 and repressive histone modification of H3K27me3 are potential factors for enriching genomic functional interactions.

In this study, we used A549 lung cancer cells and noncancerous BEAS-2B epithelial cells as model systems and applied long-read ChIA-PET [24] to map global chromatin interactions associated with different factors, including RNA polymerase II (RNAPII), CCCTC-binding factor (CTCF), EZH2, and H3K27me3. These factors were selected as CTCF represents a classic structural protein in the 3D genome, RNAPII is associated with active gene transcription, and EZH2 and H3K27me3 are associated with repressive chromatin [20], [23], [25], [26]. By studying the patterns and functions of different categories of interactions and comparing the differences in interactions between lung cancer and noncancerous cells, we discovered the mechanisms of dysregulation at the 3D genome level, providing new insights into the transcriptional regulation and genomic characteristics of human lung cancer.

## Results

### Chromatin interactions are mediated by multiple factors in A549 lung cancer cell line

**Figure 1:**
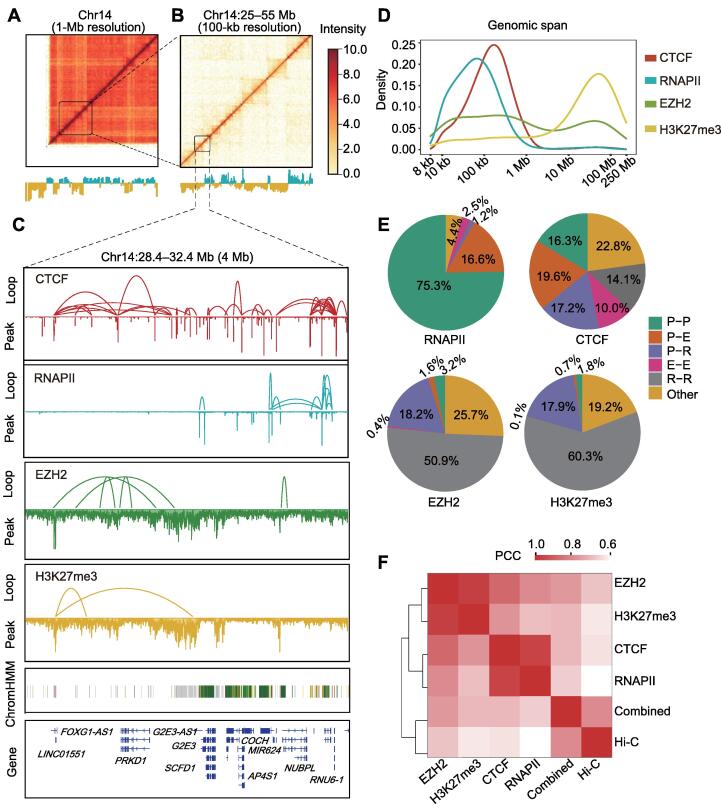
**ChIA-PET interactions mediated by four different factors in A549 lung cancer cell line** **A.** Combined chromatin interaction heatmap (1-Mb resolution) and A/B compartments of chromosome 14 in A549 cell line. The combined ChIA-PET interaction heatmap was based on CTCF, RNAPII, EZH2, and H3K27me3 ChIA-PET data. Lower panels show A (blue) and B (yellow) compartments calculated from principal component analysis using combined ChIA-PET data. **B.** Combined chromatin interaction heatmap (100-kb resolution) and A/B compartments at 25–55-Mb regions of chromosome 14. **C.** Loop and peak views of ChIA-PET data in a 4-Mb region on chromosome 14. For each data track, loop view is at the top, and peak view is at the bottom. Chromatin state annotation by ChromHMM was obtained from the NIH Roadmap Epigenomics Mapping Consortium [57]. **D.** Different genomic spans of loops in ChIA-PET associated with CTCF, RNAPII, EZH2, and H3K27me3. **E.** Pie charts of different interaction categories mediated by four factors. **F.** Heatmap of PCCs between individual-factor mediated ChIA-PET interactions for CTCF, RNAPII, EZH2, H3K27me3, combined data, and Hi-C data. ChIA-PET, chromatin interaction analysis by paired-end tag sequencing; CTCF, CCCTC-binding factor; RNAPII, RNA polymerase II; EZH2, enhancer of zeste homolog 2; H3K27me3, histone 3 lysine 27 trimethylation; Hi-C, high-throughput chromosome conformation capture; Chr, chromosome; NIH, National Institutes of Health; P, promoter; E, enhancer; R, repressor; PCC, Pearson correlation coefficient.

To investigate the 3D genome architecture and its regulatory function in lung cancer, we performed long-read ChIA-PET associated with CTCF, RNAPII, EZH2, and H3K27me3 in the lung cancer cell line A549 and noncancerous cell line BEAS-2B. These different chromatin interactomes should help clarify the 3D genome of the A549 lung cancer cell line. In total, we obtained ∼ 375 million uniquely mapped paired-end reads and ∼ 283,000 chromatin loops from six ChIA-PET libraries (Table S1). The correlations of biological replicates are shown in Figure S1, and both contact matrices and visible clusters for each factor-mediated ChIA-PET showed excellent reproducibility. We then combined interaction pairs of the four factors and constructed a contact heatmap at the chromosomal level. The 1-Mb and 100-kb resolution contact heatmaps of chromosome 14 showed chromosome interaction patterns, allowing identification of the A/B compartments and TADs (Figure 1A and B). The binding sites and chromatin interactions associated with each factor are shown in Figure 1C. We found that the EZH2- and H3K27me3-associated interaction loops mainly appeared between their broad peaks and were primarily located in repressive regions, whereas the RNAPII-associated interaction loops were mainly located in active regions. Thus, we speculated that EZH2- and H3K27me3-associated interaction loops occupy different positions on the genome than RNAPII-associated interaction loops, and their positional distributions may be related to genomic activity.

We plotted the span distributions of the loops mediated by the four factors (Figure 1D). In general, the RNAPII loops showed the shortest spans, and the loop span with the highest density was less than 100 kb. The CTCF loops showed the highest span density at slightly over 100 kb. The EZH2 loops showed two peaks in span density, with the shorter one less than 1 Mb and the longer one between 10 Mb and 100 Mb. The H3K27me3 loops showed the highest span density of between 10 Mb and 100 Mb. Jodkowska et al. [27] observed a similar span distribution of chromatin interactions from promoter capture Hi-C, but without associated proteins reported in their study.

Subsequently, we described the distribution of interactions associated with different factors on gene regulatory elements. As shown in Figure 1E, significant differences were found in pairs of regulatory elements between these factors, especially in terms of the proportion of promoter-involved interactions. Of the RNAPII-mediated loops, 92% were promoter–promoter or promoter–enhancer loops. In comparison, only a small proportion (5% for EZH2 and 2% for H3K27me3) of active promoter-centered interactions was organized by EZH2 and H3K27me3. For the two repressive factors, most of the interactions were repressor-associated interactions (69% for EZH2 and 78% for H3K27me3). Moreover, the loops associated with CTCF were mostly evenly distributed in the different categories, confirming the viewpoint that CTCF is more involved in structural formation and maintenance than in gene regulation.

We then calculated the Pearson correlation coefficients (PCCs) of the contact matrices mediated by the four factors and Hi-C contact matrices (Figure 1F). The EZH2 and H3K27me3 contact matrices showed high similarity, and the CTCF and RNAPII contact matrices showed high similarity. The combined contact matrices of the four factors were highly similar to Hi-C, suggesting that the combined interactomes of these factors reflected the whole-genome interactions observed with Hi-C. Therefore, comprehensive analysis of chromatin interactions associated with different factors can provide additional details of distinct features on the 3D genome architecture and clarify the regulatory relationship of the A549 lung cancer cell line.

### High-resolution chromatin interaction analysis can reveal hierarchical genomic structures

**Figure 2:**
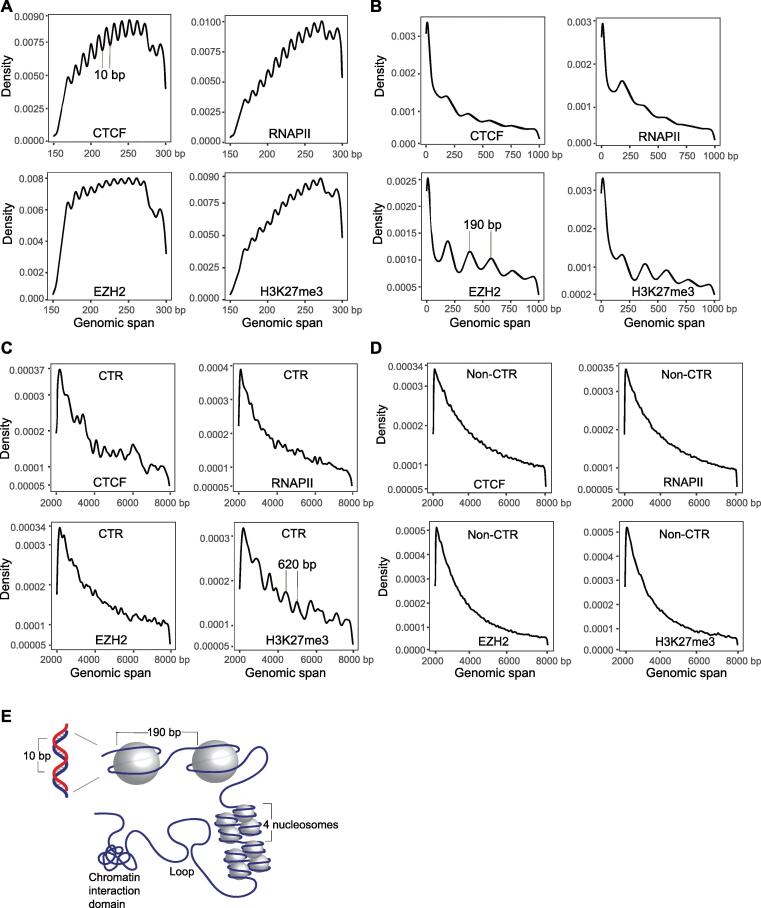
**Hierarchical 3D genome structures obtained by ChIA-PET data** **A.** Distribution of PETs spanning 150 bp to 300 bp in ChIA-PET self-ligation data. X-axis represents PET spans in bp. The 10-bp period may represent DNA double-helix turn. **B.** Distribution of PETs spanning 0 bp to 1 kb. The 190-bp span may represent mononucleosome structure. **C.** Distribution of PETs spanning 2 kb to 8 kb. Spans of 400–700 bp representing tetranucleosomes can be found in CTRs, especially in H3K27me3 ChIA-PET data. We defined CTRs as regions with overlapping H3K9me3 and H3K4me3 ChIP-seq peaks. **D.** There were no prominent periodic peaks in density curves in non-CTRs. **E.** Model of DNA hierarchical structures revealed by A549 ChIA-PET data. 3D, three-dimensional; CTR, chromatin transitional region; PET, paired-end tag; ChIP-seq, chromatin immunoprecipitation with sequencing.

The ChIA-PET method uses sonication for chromatin fragmentation and antibodies for factor enrichment, which can increase the resolution of interaction maps and functional elements. Based on the ChIA-PET data, we observed hierarchical genomic structures at different scales, including A/B compartments and TAD-like structures (Figure 1A and B), as well as finer structures. By examining the span of the paired-end reads, we observed that DNA distance distribution was periodic. First, we detected 10-bp (Figure 2A) and 190-bp periods (Figure 2B), reflecting the DNA double-helix turn and single-nucleosome structure, respectively, thus indicating that the interaction positions were not randomly distributed around the nucleosomes. Second, we observed 400–700-bp periods in the chromatin transitional regions [CTRs; located between active and inactive regions, defined as the center 2 kb of the overlapping regions of the H3K9me3 and H3K4me3 chromatin immunoprecipitation with sequencing (ChIP-seq) peaks] in the ChIA-PET data associated with H3K27me3 (Figure 2C). Such periods were not observed in the non-CTRs (Figure 2D). The 3–4 nucleosome structure in the CTRs may be unique, as CTRs are the boundaries between heterochromatin and euchromatin. Nucleosomes at CTRs can reduce the turnover rate and tend to form tetranucleosomes, which may be a basic structure in chromosome fiber assembly [28]. To validate our observations based on ChIA-PET methods, we analyzed HeLa CTCF ChIA-PET (GEO: GSE72816), GM12878 CTCF ChIA-PET (GEO: GSE72816), and mouse embryonic stem cell (mESC) SUZ12 ChIA-PET (GEO: GSE120393), and also observed the 10-bp and 190-bp periods (Figure S2A–C), thus validating our observations. Therefore, high-resolution ChIA-PET data showed hierarchical 3D genome structures at different scales (Figure 2E).

To check whether similar hierarchical chromatin structures can be observed from Hi-C and *in situ* Hi-C followed by chromatin immunoprecipitation (HiChIP) methods, we used A549 Hi-C data (ENCODE: ENCSR662QKG), IMR-90 Hi-C data (ENCODE: ENCSR852KQC), HeLa CTCF HiChIP data (GEO: GSE108869), and GM12878 CTCF HiChIP data (GEO: GSE115524) to conduct the same analyses. We observed A/B compartments, TADs, and loops from these datasets. However, the patterns of the 10-bp, 190-bp, and 400–700-bp periods were not observed in Hi-C and HiChIP data (Figure S2D–G).

With this result in mind, we considered the potential reason for observing the 10-bp and 190-bp period patterns in the ChIA-PET data, but not in the Hi-C and HiChIP data. Both the Hi-C and HiChIP methods are based on enzymatic digestion, and the span of paired-end reads is mainly determined by enzyme cutting sites. In contrast, the ChIA-PET method uses sonication to fragment chromatin, and the breakpoints are between nucleosomes. This may explain the differences in the observation of the 10-bp and 190-bp periods.

### EZH2- and H3K27me3-mediated chromatin interactions present distinct patterns and transcriptional activities from RNAPII-mediated chromatin interactions

**Figure 3:**
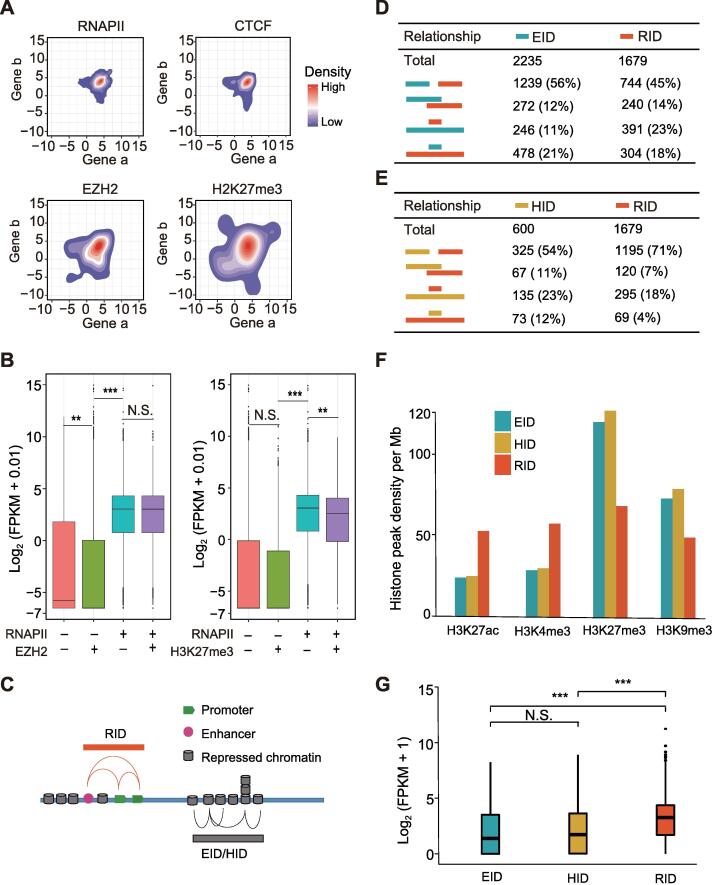
**Characterization of chromatin interactions mediated by four factors** **A.** Contour plots showing log_2_-transformed RNA-seq FPKM values for promoter–promoter interacting genes in A549 cells. Gene pairs with RNAPII- and CTCF-mediated interactions show concentrated high gene expression levels, whereas gene pairs with EZH2- and H3K27me3-mediated interactions show more dispersed gene expression levels. **B.** Boxplots showing gene expression with or without interactions mediated by different factors. *P* value was determined using one-sided Mann–Whitney *U* test. “+”, genes with loops mediated by this factor on gene promoter (±2-kb regions around TSS). “−”, no loops of this factor on gene promoter. **C.** Model of interaction domains (defined as a genomic region spanned by continuously connected loops mediated by specific factors). **D.** Table showing relative positions and proportions of EIDs and RIDs. Proportions of independent, partially intersecting, and inclusive relationships of different interaction domains are shown. **E.** Table showing relative positions and proportions of HIDs and RIDs. **F.** Densities of histone modification peaks per Mb in EIDs, HIDs, and RIDs. **G.** Boxplots showing gene expression levels in EIDs, HIDs, and RIDs. *P* value was determined using one-sided Mann–Whitney *U* test. **, *P* < 0.01; ***, *P* < 0.001; N.S., no significant difference (*P* > 0.05). EID, EZH2-mediated interaction domain; HID, H3K27me3-mediated interaction domain; RID, RNAPII-mediated interaction domain; RNA-seq, RNA sequencing; FPKM, fragments per kilobase of exon model per million mapped fragments; TSS, transcription start site.

Interactions mediated by RNAPII and CTCF have been described in several studies [25], [26], and RNAPII-mediated promoter–promoter interactions can link gene pairs with strong coexpression patterns [26]. However, the characteristics and profiles of repressive interactions in lung cancer remain to be studied. Here, we determined whether genes with EZH2- or H3K27me3-mediated promoter–promoter interactions are also transcriptionally coordinated. RNA sequencing (RNA-seq) data indicated that most of the paired genes with RNAPII-mediated promoter–promoter interactions showed high simultaneous expression, with a small range in variation. The paired genes with CTCF-mediated promoter–promoter interactions exhibited a slightly larger range of variation in expression levels. In contrast, paired genes with EZH2- or H3K27me3-mediated promoter–promoter interactions displayed the largest range of variation in expression levels (Figure 3A). There were more paired genes with low expression in the repressive interactions than in the RNAPII-mediated interactions. To further assess the coordinated transcription of paired genes across different conditions, we performed Pearson correlation analysis using The Cancer Genome Atlas (TCGA) lung adenocarcinoma (LUAD) RNA-seq data. Results indicated that genes involved in the RNAPII-mediated promoter–promoter interactions were highly correlated, whereas those involved in the promoter–promoter interactions mediated by EZH2 and H3K27me3 were lowly correlated (Figure S3A). These analyses indicate that most gene pairs involved in the RNAPII-mediated promoter–promoter interactions tend to be highly cooperatively transcribed. In contrast, gene pairs involved in EZH2- or H3K27me3-mediated promoter–promoter interactions tend to be weakly (or not at all) cooperatively transcribed.

In previous ChIP-seq or genome-wide association study (GWAS) SNP analyses, regulatory elements are assumed to regulate their nearest genes [29], [30]. However, this may not be true in general. We observed that more than 64% of enhancers and 87% of repressors did not interact with their nearest promoters, instead bypassing several intervening genes to reach their target promoters (Figure S3B). In addition, we observed that genes involved in proximal promoter–enhancer interactions had significantly higher expression levels than genes involved in distal promoter–enhancer interactions (*P* < 1.2E−05, Mann–Whitney *U* test), and genes involved in proximal promoter–repressor interactions had significantly lower expression levels than genes involved in distal promoter–repressor interactions (*P* < 4.6E−07, Mann-Whitney *U* test) (Figure S3C). Therefore, we speculated that gene transcription levels depend on the genomic distances of promoter–enhancer and promoter–repressor interactions to some extent.

We further investigated the effects of RNAPII- and EZH2-mediated loops on gene transcription. Results indicated that genes in the EZH2-mediated loop anchors, but not in the RNAPII-mediated loop anchors, had the lowest expression levels, with levels lower than those of genes in other loop anchors (*P* < 2.2E−16, Mann–Whitney *U* test) (Figure 3B). In contrast, genes in the RNAPII-mediated loop anchors showed high expression levels. A similar trend was found when comparing the expression levels of genes with RNAPII-mediated and H3K27me3-mediated interactions (Figure 3B). We also found that promoters bound to EZH2/H3K27me3-mediated loop anchors with peaks were more repressed than promoters only bound to peaks, only bound to loop anchors, or neither (Figure S3D). These results suggest that EZH2- and H3K27me3-mediated loops may further repress the expression of the target genes.

To characterize the spatial relationships between RNAPII-, EZH2-, and H3K27me3-associated chromatin topologies, we defined chromatin interaction domains based on loop connectivity and contact frequency, *i.e.*, RNAPII-mediated interaction domain (RID), EZH2-mediated interaction domain (EID), and H3K27me3-mediated interaction domain (HID) (Figure 3C, Figure S3E). In total, we identified 1679 RIDs, 2235 EIDs, and 600 HIDs. Most RIDs were isolated from HIDs (71%) and EIDs (45%), whereas 23% of RIDs were contained in EIDs and 18% of RIDs were contained in HIDs (Figure 3D and E). Epigenomic features are also reportedly associated with 3D genome architecture [31], [32]. Here, we investigated the epigenomic marks and transcriptional activities of different chromatin interaction domains. We found that EIDs and HIDs exhibited lower densities of active histone marks (H3K27ac and H3K4me3) and higher densities of inactive histone marks (H3K27me3 and H3K9me3) than RIDs (Figure 3F). In addition, EIDs and HIDs contained fewer active genes (Figure S3F) and exhibited lower transcriptional activities (*P* < 2.2E−16, Mann-Whitney *U* test) (Figure 3G) than RIDs. Comparing the A and B compartments identified in the Hi-C data, we found that 75% of RIDs were located in the A compartments, with 6% of RIDs located in the B compartments, whereas the B compartments contained more EIDs and HIDs than the A compartments (Figure S3G). These findings suggest that the interactions associated with different factors exhibit distinct distributions and coexpression features across the genome, and that factor-specific chromatin interaction domains exhibit distinct epigenomic properties that are highly consistent with transcriptional activity.

### High-resolution loops map whole-genome regulatory relationship of lung cancer-related genes and SNPs

**Figure 4:**
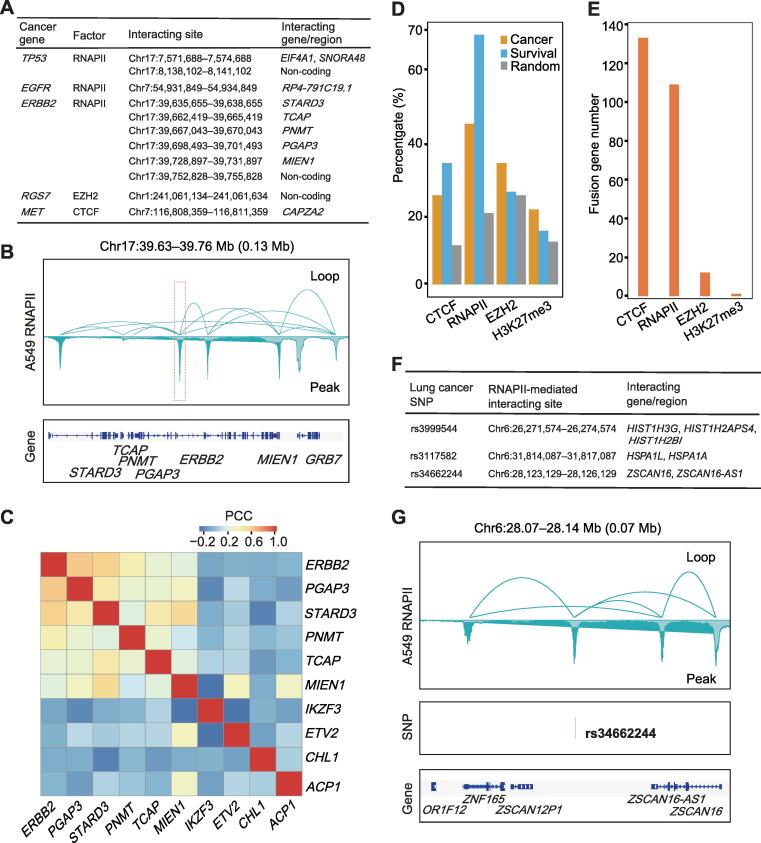
**ChIA-PET interaction pairs show regulatory relationship of cancer risk-related genes and SNPs** **A.** Typical regions or genes interacting with lung cancer-related genes. If a gene promoter was located in the interacting region, it was defined as an interacting gene. Non-coding means that the interacting region is an intergenic region far from the gene body. **B.** RNAPII-mediated interactions around oncogene *ERBB2*. **C.** PCCs between genes in TCGA LUAD tumor samples. *ERBB2* is a lung cancer-related gene. *STARD3*, *TCAP*, *PNMT*, *PGAP3*, and *MIEN1* are linked with *ERBB2* by RNAPII-mediated loops. They are highly coexpressed with *ERBB2*. *IKZF3* is a gene near *ERBB2* and is not linked with *ERBB2*. *ETV2* is an inter-chromosome linking gene related to *ERBB2*. *CHL1* and *ACP1* are two randomly selected genes located on other chromosomes without interactions with *ERBB2* and were used as negative controls. **D.** Proportions of cancer- and survival-related genes involved in remote interactions mediated by four factors. “Random” represents 100 genes randomly selected in the genome, and average of three randomized trials is shown. **E.** Number of fusion transcripts overlapping with interaction loops associated with different factors. In total, 854 gene pairs from TCGA fusion transcripts and 854 random gene pairs were analyzed. Three trials of random gene pairs did not overlap with any loops. **F.** Selected regions or genes interacting with risk SNPs in lung cancer. **G.** Example of RNAPII-mediated interactions linking SNP rs34662244 in an intergenic region in chromosome 6 and its target genes. According to ChIA-PET data, *ZSCAN 16*, *ZSCAN 16-AS1*, and *ZNF165* are target genes of this SNP. SNP, single nucleotide polymorphism; TCGA, The Cancer Genome Atlas; LUAD, lung adenocarcinoma.

Genomic interactions can reveal the spatial proximity and regulatory events of genomic sites [26], [33], [34]. Interaction mapping of cancer-related genes may increase our understanding of the regulatory elements of these genes. ChIA-PET loops can directly link distal elements to the promoters of target genes to yield regulatory information and can accurately identify specific transcription start sites (TSSs). Here, we adopted 203 oncogenes and tumor suppressor genes from the Network of Cancer Genes (NCG) [35] and the top 100 survival-related genes from the Gene Expression Profiling Interactive Analysis (GEPIA) database [36]. We then identified genes and non-coding regions that interact with lung cancer-related genes using high-resolution interaction data (Tables S2 and S3). Several typical lung cancer-related genes exhibited interactions mediated by CTCF, EZH2, H3K27me3, and especially RNAPII (Figure 4A; Tables S2 and S3), suggesting a regulatory relationship between the associated chromatin interactions and these genes.

As seen in Figure 4B, the oncogene *ERBB2* showed several strong RNAPII-mediated interactions with nearby genes, such as *PGAP3*, *STARD3*, and *PNMT*. Compared with neighboring or distant genes not in the interaction domain, genes with *ERBB2* interactions demonstrated obvious coexpression patterns in the LUAD tumor samples (Figure 4C), whereas similar coexpression patterns were not observed in normal samples (Figure S4A). Thus, the coexpression pattern between *ERBB2* and other genes, such as *PGAP3*, *STARD3*, and *PNMT*, may be a marker of cancer, and site mutations in the interaction anchors or dysregulation in interactions related to *ERBB2* may be a new target for cancer studies.

Another example from interaction mapping was the survival-related gene *DKK1*, which was not involved in EZH2-meditated interactions and showed higher expression in the A549 cell line than in BEAS-2B cell line, with log_2_ fold change > 1 and adjusted *P* < 0.01 (Figure S4B; Table S5). Interestingly, lung cancer-related genes were more enriched in the RNAPII- and CTCF-meditated interactions than randomly selected genes (*P <* 2.87E−07, chi-square test; Figure 4D). This enrichment suggests that mapping the interactomes of these genes may facilitate studies on the potential carcinogenic function of these cancer-related interactions in lung cancer.

In lung cancer samples, tumor-specific fusion transcripts are suggested to disrupt normal gene function or activate proto-oncogenes, thus driving tumorigenesis [37], [38], [39]. We assessed the TumorFusions database list [40] of TCGA LUAD samples to determine whether long-range interactions exist in the spatial genome between the fusion gene pairs. Among the 854 pairs of host fusion genes, more than 100 exhibited interactions mediated by RNAPII or CTCF, whereas only a few pairs showed interactions mediated by EZH2 or H3K27me3. As a control, among 854 randomly selected pairs of expressed genes, none were involved in the interactions. Thus, the pairs of host fusion genes were also highly enriched in CTCF- and RNAPII-meditated interactions (*P <* 2.2E−16, chi-square test; Figure 4E). These results suggest a positive relationship between TCGA fusion transcripts and spatial contacts, in which interaction loops may be potential facilitators of fusion transcripts.

We also mapped the interacting sites of high-risk SNPs in lung cancer (Figure 4F; Table S4). The lung cancer risk SNP rs34662244 was located in the anchor of two enhancer–promoter interaction clusters with target genes *ZNF165*, *ZSCAN16*, and *ZSCAN16-AS1* (Figure 4G). These interaction clusters suggest a potential role of this non-coding SNP in regulating target genes over a long distance. Moreover, based on a whole-gene expression comparison of TCGA LUAD tumor samples and normal samples, we found that a large proportion of genes targeted by SNPs through RNAPII-mediated interactions had higher expression levels in lung cancer samples than in normal samples (*P* = 0.004, Wilcoxon test; Figure S4C), suggesting a potential cancer driver function of SNP-associated genes. Therefore, our high-resolution chromatin interaction landscape may provide important regulatory information on lung cancer-related genes and SNPs.

### Associations between altered chromatin interactions and dysregulation of oncogenes and tumor suppressor genes

As RNAPII- and EZH2-mediated interactions showed significant regulation of gene expression, we analyzed whether abnormal expression of oncogenes or tumor suppressor genes is related to abnormal interaction patterns in lung cancer. We conducted joint analysis of differential gene expression and differential interactions between the lung cancer cell line A549 and noncancerous cell line BEAS-2B (Figure S5A and B). We found that 74% of genes involved with RNAPII-mediated interactions in A549 cells were also involved with RNAPII-mediated interactions in BEAS-2B cells, whereas only 22% of genes involved with EZH2-mediated interactions in A549 cells were also involved with EZH2-mediated interactions in BEAS-2B cells. The differentially expressed and A549-specific anchor genes were enriched in the Gene Ontology (GO) biological process term “anterior/posterior pattern specification”. Genes enriched in this term were mainly lung cancer-related HOXB gene clusters on chromosome 17 and were involved in RNAPII-mediated interactions, with higher expression in A549 cells than in BEAS-2B cells (Figure S5C). The differentially expressed and BEAS-2B-specific anchor genes were significantly enriched in “DNA binding” (Figure S5D), and most belonged to the ZNF gene family and functioned as transcription factors. We speculated that the ZNF gene family may play a role in lung cancer tumorigenesis.

**Figure 5:**
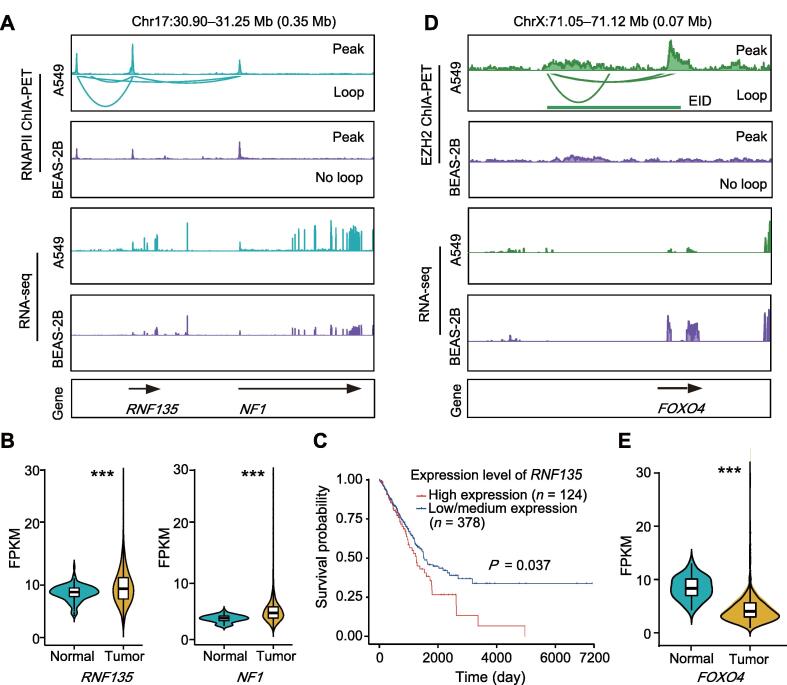
**Differential interactions are associated with abnormal expression of oncogenes or tumor suppressor genes in lung cancer** **A.** Differential RNAPII-mediated interactions associated with *RNF135* and *NF1* in lung cancer cell line A549 and noncancerous cell line BEAS-2B. RNA-seq tracks show that both *RNF135* and *NF1* have higher expression levels in A549 cells than in BEAS-2B cells. **B.** Violin plots overlaid with boxplots showing the distribution of *NF1* and *RNF135* mRNA expression levels in a large set of LUAD tumor tissues and normal tissues from TCGA database. *P* value was determined using one-sided Mann–Whitney *U* test. **C.** Overall survival curve showing that overexpression of *RNF135* is correlated with poor outcome. This figure was made using online portal UALCAN [45]. The red survival curve is for patients with *RNF135* expression in the highest quartile, and the blue curve is for other samples with lower *RNF135* expression. **D.** Differential EZH2-mediated interactions associated with *FOXO4* in lung cancer cell line A549 and noncancerous cell line BEAS-2B. Based on RNA-seq tracks, *FOXO4* showed lower expression in A549 cells than in BEAS-2B cells. **E.** Violin plots overlaid with boxplots showing the distribution of *FOXO4* mRNA expression levels in a large set of LUAD tumor tissues and normal tissues from TCGA database. *P* value was determined using one-sided Mann-Whitney *U* test. ***, *P* < 0.001. mRNA, messenger RNA.

Among genes associated with altered interactions, *RNF135* and *NF1* are two adjacent genes located on chromosome 17 [41]. The *NF1* gene represses the RAS signaling pathway, and its mutation or abnormal expression may lead to lung cancer [42], [43]. Studies have suggested that *RNF135* may exhibit oncogenic functions in glioblastoma [44]. Here, based on RNA-seq differential expression analysis, *NF1* and *RNF135* showed significantly higher expression in the A549 cells than in the BEAS-2B cells (log_2_ fold change > 1 and adjusted *P* value < 0.01; Table S5). There was a strong RNAPII-mediated promoter–promoter interaction cluster between these two genes in the A549 cells (Figure 5A), which was not observed in the BEAS-2B cells. Promoter–promoter interaction seems to increase the expression of this gene pair. In TCGA database analysis, both *NF1* and *RNF135* showed higher expression levels in the LUAD tumor samples than in the normal samples (*P* = 1.2E−10 for *NF1* and *P* = 8.8E−04 for *RNF135*, Mann–Whitney *U* test; Figure 5B). In the survival analysis, *RNF135* overexpression indicated poor outcome in patients (Figure 5C).

Abnormal repression of tumor suppressor genes is another crucial aspect of cancer [23]. In our study, the repression of several tumor suppressor genes was associated with EZH2-related loops. For example, *FBLN1* and *FOXO4*, well-known lung cancer suppressor genes [46], [47], [48], showed significantly lower expression in the A549 cells than in the BEAS-2B cells (log_2_ fold change < −1 and adjusted *P* value < 0.01; Table S5). EZH2-mediated interactions were associated with these two genes in the A549 cells, but not in the BEAS-2B cells (Figure 5D, Figure S6A). The two genes also exhibited lower expression in LUAD samples than in normal samples (*P <* 2.2E−16 for *FBLN1* and *FOXO4*, Mann–Whitney *U* test; Figure 5E, Figure S6B). These results suggest that chromatin interactions are closely related to the maintenance of lung cancer-related gene expression.

**Figure 6:**
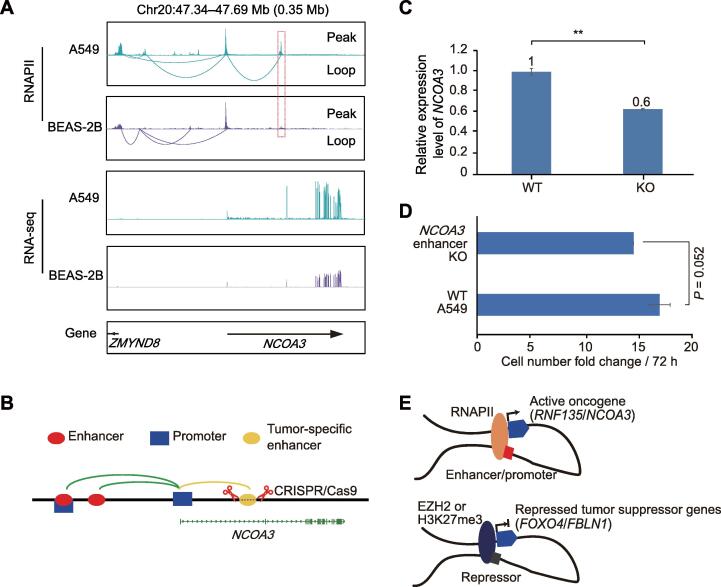
**KO of A549-specific anchor can reduce abnormal expression of oncogene *NCOA3*** **A.** Differential RNAPII-mediated interactions associated with oncogene *NCOA3* in lung cancer cell line A549 and noncancerous cell line BEAS-2B. There is an A549-specific interaction linking intron enhancer and promoter of *NCOA3*. RNA-seq tracks show that *NCOA3* has a higher expression level in A549 cells than in BEAS-2B cells. **B.** Schematic of KO of interaction anchor in *NCOA3*. **C.***NCOA3* gene expression levels were detected by qRT-PCR. Three biological RNA samples of *NCOA3* KO cells were used. *P* value was determined using two-sided paired *t*-test. **, *P* < 0.01. **D.** Cell growth rate indicated by cell number fold change over 72 h. Fold change in cell growth was calculated in three sets of 6-well plates. *P* value was determined using two-sided paired *t*-test. **E.** Models show functions of active chromatin interactions in the regulation of oncogenes (upper) and repressive chromatin interactions in the regulation of tumor suppressor genes (lower). KO, knockout; WT, wild-type; qRT-PCR, quantitative real-time polymerase chain reaction; CRISPR, clustered regularly interspaced short palindromic repeats.

To test whether A549-specific interaction anchors can function as oncogenic regions, we performed clustered regularly interspaced short palindromic repeats (CRISPR)/Cas9-targeted knockout (KO) of the A549-specific anchors to verify the effects of dysregulated interactions on cancer gene expression. *NCOA3* is a well-studied oncogene that is up-regulated in many cancers [49], [50]. In this study, *NCOA3* expression was much higher in the A549 cells than in the BEAS-2B cells (log_2_ fold change > 1 and adjusted *P* value < 0.01; Table S5). In the A549 cells, there was a specific RNAPII-mediated enhancer–promoter interaction cluster between the promoter of *NCOA3* and an enhancer located in the first intron of *NCOA3* (Figure 6A), which did not exist in the BEAS-2B cells. We knocked out the 1-kb enhancer region in the A549 cells with CRISPR (Figure 6B) and characterized gene expression changes. Results showed that *NCOA3* expression was significantly lower in the KO A549 cells than in the wild-type (WT) A549 cells (*P* < 0.01, two-sided paired *t*-test; Figure 6C). *NCOA3* is known to have a positive effect on tumor cell growth [49], [50]. Our study indicated that KO of the A549-specific *NCOA3* enhancer showed a tendency of reduced cell growth rate (Figure 6D).

Based on the aforementioned results, we proposed two models to describe the effects of altered interactions in lung cancer on activating oncogenes and inhibiting tumor suppressor genes, as shown in Figure 6E. In the lung cancer cells, some oncogenes were activated by active factor-associated interactions, and some tumor suppressor genes were repressed by repressive factor-associated interactions. These results suggest that dysregulated chromatin interaction patterns may be an important aspect of tumorigenesis, thereby highlighting the importance of normal interactions in cells.

## Discussion and conclusion

Chromatin interactions play important roles in gene transcription regulation and other biological functions. However, few studies have been conducted on repressive chromatin interactions and their relationship with active chromatin interactions. In this study, we mapped the high-resolution genome-wide interactomes mediated by EZH2, H3K27me3, RNAPII, and CTCF in lung cancer A549 cells, representing repressive chromatin interactions, active chromatin interactions, and structural chromatin interactions, respectively. We demonstrated that different types of interactions exhibited different distributions and regulatory functions in the genome. In most cases, repressive and active interactions were distributed in different genomic regions, and the repressive interactions primarily linked repressive elements to silence or repress gene transcription.

Our high-resolution ChIA-PET data revealed 10-bp and 190-bp signals, representing the DNA double helix turn and mononucleosome distance, respectively. Interestingly, the 3–4 nucleosome signals in the facultative heterochromatin hinted at structures mentioned in previous studies in the context of chromosome fiber assembly [28].

We used high-resolution chromatin interactions to map the whole-genome regulatory relationship of lung cancer-related genes and SNPs. As tumor-related genes and fusion transcripts were enriched in the interaction loops, we speculated on the central role of long-range interactions in tumorigenesis. We further described how altered interactions can dysregulate the expression of cancer-related genes. The high-resolution 3D landscape generated from this study may expand our understanding of the lung cancer genome.

## Materials and methods

### Cell culture

The A549 cell line (accession No. CCL-185) was purchased from the American Type Culture Collection (ATCC) and was authenticated by the GENEWIZ Company. The BEAS-2B cell line was donated by Dr. Honglin Jin’s laboratory at the Cancer Center of Union Hospital, Tongji Medical College, Huazhong University of Science and Technology, China. The A549 and BEAS-2B cell lines were cultured at 37 °C under 5% CO_2_ in air. The A549 cells were cultured in Ham’s F12K medium (Catalog No. 21127022, Gibco, Grand Island, NY) supplemented with 10% fetal bovine serum (FBS; Catalog No. 10099141, Gibco), 0.1 mM non-essential amino acids (Catalog No. 11140050, Gibco), and 1× penicillin/streptomycin (Catalog No. 15140163, Gibco). The BEAS-2B cells were cultured in RPMI 1640 (Catalog No. 11875101, Gibco) supplemented with 10% FBS and 100 U/ml penicillin/streptomycin. For both cell lines, the medium was changed every other day. The cells were passaged by trypsin digestion three times per week.

### Long-read ChIA-PET

For long-range interaction analysis of the A549 and BEAS-2B cells, we used long-read ChIA-PET as described previously [24] with some minor modifications. The cells were resuspended using trypsin digestion and then dual cross-linked with 1.5 mM ethylene glycol bis (succinimidyl succinate; Catalog No. 21565, ThermoFisher Scientific, Waltham, MA) for 40 min and with 1% formaldehyde (Catalog No. F8775, Sigma-Aldrich, St Louis, MO) for 10 min at room temperature. The cells were lysed, and chromatin was fragmented into 1–5-kb fragments by sonication (high level, 33 cycles, 30 s ON, 50 s OFF) using the sonication device (Bioruptor Plus, Diagenode, Belgium). Chromatin immunoprecipitation was used to enrich the complex fragments with magnetic beads of protein G (Catalog No. 10009D, ThermoFisher Scientific) and 60–100 µg of antibodies against RNAPII (Catalog No. SC-56767, Santa Cruz Biotechnology, Dallas, TX), EZH2 (Catalog No. 5246, Cell Signaling Technology, Danvers, MA), H3K27me3 (Custom-made, ABclonal, Wuhan, China), and CTCF (Catalog No. A1133, ABclonal). The beads were then washed, and DNA blunt ends were prepared with A tails. Bridge linkers were used to ligate the proximal DNA ends in a 1.8-ml reaction system using T4 DNA ligase (Catalog No. EL0013, ThermoFisher Scientific). The DNA extraction and library construction steps were the same as used in the previous protocol [24]. The libraries were paired-end sequenced (2 × 150 bp) using the Illumina HiSeq X Ten system (HiSeq X10, Illumina, San Diego, CA).

### Total RNA-seq

Two A549 RNA-seq replicates were generated. Total RNA was extracted from one million cells using RNeasy columns (Catalog No. 74104, QIAGEN, Hilden, Germany). Ribosomal RNA (rRNA) was depleted using an rRNA Depletion Kit (Catalog No. E7400, New England Biolabs, Ipswich, MA), and then strand-specific libraries were constructed using the NEBNext Ultra II RNA Library Prep Kit (Catalog No. E7645L, New England Biolabs) for Illumina. Paired-end (2 × 150 bp) sequencing of libraries was conducted using the Illumina HiSeq X Ten system (HiSeq X10, Illumina).

### **CRISPR/Cas9-mediated KO of *NCOA3*-specific enhancer**

For the KO system, we applied chromatin fragment deletion as described previously with some modifications [51]. Single-guide RNA (sgRNA) templates were constructed and incorporated into pGL3-U6-sgRNA-PGK-Puromycin plasmids. The A549 cells were cultured to approximately 60% confluence and transfected with Lipofectamine 3000 (Catalog No. L3000001, ThermoFisher Scientific) in a 6-well plate with 2 μg of pcDNA3.1-Cas9 and 1.5 μg of sgRNA plasmids for the upstream target and 1.5 μg of sgRNA plasmids for the downstream target. Puromycin was added 2 days later to a final concentration of 1.2 μg/ml. The cells were then diluted and plated in 15-cm plates and cultured for 5 days to isolate single clones. For KO of the *NCOA3*-specific enhancer, we designed sgRNA sequences: upstream 1, 5′-ATAGAATTGCAACCTCATGGAGG-3′; upstream 2, 5′-CAGTTGTACCTACTGCCAAATGG-3′; downstream 1, 5′-CCCCCTTTGGCTGAGATAAATGG-3′; downstream 2, 5′-GTCTAGCACAATGTGGCACATGG-3′.

### RNA expression analysis by qRT-PCR

Here, qRT-PCR was conducted on a RT PCR detection system (CFX Connect, Bio-Rad, Hercules, CA). We used Genious 2X SYBR Green Fast qPCR Mix (Catalog No. RM21203, ABclonal) for qRT-PCR. Total RNA was extracted from one million cells by RNeasy columns (Catalog No. 74104, QIAGEN). We used 2 µg of RNA to perform reverse transcription with random primers using TransScript One-Step gDNA Removal and cDNA Synthesis SuperMix (Catalog No. AT311-02, TransGen Biotech, Beijing, China). The qRT-PCR was then performed using primers (forward, 5′-GGACCTGGTTAACACAAGTG-3′; reverse, 5′-GTCCAGGAAACTCCATTAACTG-3′) for *NCOA3* messenger RNA (mRNA).

### Analysis of ChIA-PET data

Long-read ChIA-PET sequence data were analyzed using a modified ChIA-PET Tool pipeline (version 3) [52]. Briefly, after trimming the linkers, the sequences flanking the linker were mapped to the human reference genome (hg38) using BWA-MEM (version 0.7.7) [53], and only uniquely mapped (mapping qualities ≥ 30) paired-end tags (PETs) were retained. Each PET was categorized as either a self-ligation PET (two ends of the same DNA fragment, with a genomic span less than 8 kb) or an inter-ligation PET (two ends from two different DNA fragments in the same chromatin complex from different chromosomes, or from the same chromosome with a genomic span of more than 8 kb). Self-ligation PETs were used for binding site calling, and inter-ligation PETs were used for long-range interaction calling. For RNAPII and CTCF, we used centered 3-kb regions of RNAPII and CTCF ChIP-seq peaks (GEO: GSE31477 [54]) as given anchors to call interaction clusters. To obtain high-confidence interactions, we included the detected interactions only if the false discovery rate was less than 0.05 and the PET count was three or more. To evaluate the robustness of the ChIA-PET method, we analyzed the biological replicates of the ChIA-PET libraries at several different resolutions. Some analysis results are presented in Figure S1. Moreover, we transformed unique ChIA-PET mapping reads to a contact matrix using ChIA-PET2 (version 0.9.3) [55] software and normalized the matrix using the iterative correction method in HiC-Pro (version 2.11.1) [56] software. Juicer (version 1.7.6) [57] was used to calculate A/B compartments and TADs. When calculating PCCs of the contact matrices mediated by four factors and the Hi-C contact matrices, we used Hi-C data from the ENCODE database (ENCODE: ENCSR662QKG).

When comparing chromatin interactions from the lung cancer cell line A549 and noncancerous cell line BEAS-2B, we defined “altered” chromatin interactions as those specific to the A549 cell line.

### Hierarchical chromatin structure analysis

We calculated the genomic spans of the self-ligation and inter-ligation PETs and observed 10-bp and 190-bp signals, respectively. We speculated that the two signals were DNA double-helix turn and nucleosome units, respectively. To observe the tetranucleosome signal, we defined the CTRs (center 2 kb of overlapping regions of H3K9me3 and H3K4me3 ChIP-seq peaks) between euchromatin and heterochromatin. H3K9me3 and H3K4me3 ChIP-seq data were obtained from the Gene Expression Omnibus database (GEO: GSE29611). Nearly 11,003 CTRs were obtained. We choose inter-ligation PETs in CTRs and calculated their genomic spans.

### Annotation of interaction loops

We annotated loops using gene promoter, enhancer, and repressor information. The promoters were defined as the ±2-kb regions around the TSSs. Cell type-specific enhancer and repressor annotations were adopted from the A549 ChromHMM data [58]. Regions with states 6_EnhG and 7_Enh were defined as enhancers and regions with states 13_ReprPC and 14_ReprPCWk were defined as repressors. We classified loops according to the overlap of interaction anchors with promoters, enhancers, or repressors, with priority given to the promoter region. For example, we defined promoter–promoter loops as both interaction anchors overlapping with promoters, promoter–enhancer loops as one anchor overlapping with a promoter and the other anchor overlapping with an enhancer, and promoter–repressor loops as one anchor overlapping with a promoter and the other anchor overlapping with a repressor. We further categorized the promoter–enhancer and promoter–repressor loops according to enhancer/repressor locations in relation to the gene body: intra-genic proximal enhancers/repressors, which are located inside a gene body and interact with the nearest promoters; extra-genic proximal repressors/enhancers, which are located outside a gene body and interact with the nearest promoters; intra-genic distal enhancers/repressors, which are located inside a gene body, bypass nearby genes, and interact with gene promoters over long distances; and extra-genic distal enhancers/repressors, which are located outside all gene bodies, bypass nearby genes, and interact with gene promoters over long distances.

### Coexpression analysis of genes with promoter–promoter interactions by PCCs

To investigate the coexpression of genes with the CTCF-, RNAPII-, EZH2-, and H3K27me3-meditated promoter–promoter interactions defined by ChIA-PET, we calculated PCCs between expression levels of gene pairs and promoter–promoter interactions. Gene expression data of 535 LUAD samples were downloaded from the TCGA. Genes involved in promoter–promoter interactions were randomly rewired to compile a random control with the same gene background but different pairings. Randomly selected gene pairs with similar distributions of genomic span and gene density as the promoter–promoter regions were used as additional controls.

### Analysis of chromatin interaction domains

A chromatin interaction domain is a genomic region spanned by continuously connected loops mediated by specific factors. First, based on the continuous connectivity of loops, we clustered loops into candidate domains. Second, we calculated loop coverage along all chromosomes at base-pair resolution and subtracted low-coverage regions from the candidate domains. Finally, the interaction domains with genome size smaller than 10 kb were excluded, and the final EIDs, HIDs, and RIDs were obtained. When comparing histone modifications of different domains, we used ChIP-seq data (H3K27ac, H3K4me3, H3K27me3, and H3K9me3) obtained from the GEO database (GEO: GSE29611 and GSE32465).

### RNA-seq data processing

Strand-specific poly(A) RNA-seq libraries were generated and sequenced as 150-bp paired-end reads. For RNA-seq analysis, we conducted quality control using FastQC (https://www.bioinformatics.babraham.ac.uk/projects/fastqc/), and adaptor sequences were removed using Trimmomatic (version 0.32) [59]. After quality filtering, the reads were mapped to the human reference genome (hg38) by HISAT2 (version 2.1.0) [60], and gene expression was qualified by StringTie (version 1.3.4d) [61]. Differential gene expression was calculated using the DESeq2 [62] package in R. To identify significant differentially expressed genes between the A549 and BEAS-2B cell lines, we used expression level thresholds of adjusted *P* value < 0.01 and |log_2_ fold change| > 1.

### Gene expression at loop anchors

We divided genes into four categories according to whether their gene promoters were associated with RNAPII-mediated loops or H3K27me3-mediated loops: (1) genes whose promoters were only associated with RNAPII-mediated loops but not associated with H3K27me3-mediated loops; (2) genes whose promoters were associated with both RNAPII-mediated loops and H3K27me3-mediated loops; (3) genes whose promoters were neither associated with RNAPII-mediated loops nor H3K27me3-mediated loops; and (4) genes whose promoters were not associated with RNAPII-mediated loops but were associated with H3K27me3-mediated loops. Significant differences between the fragments per kilobase of exon model per million mapped fragments (FPKM) values of these different categories of genes were compared using the Mann–Whitney *U* test. Similarly, we divided genes into four categories according to whether their promoters were associated with EZH2/RNAPII-mediated loops or EZH2/RNAPII-mediated binding sites and compared expression differences between different categories.

### GO enrichment analysis

For genes in cell-specific anchors with significant differential expression, we examined enrichment in GO terms using the Database for Annotation, Visualization, and Integrated Discovery (DAVID) [63].

### TCGA RNA-seq data analysis

We downloaded gene expression data for 535 primary LUAD tumor tissues and 59 solid normal lung tissues from the TCGA database [64] and analyzed their gene expression differences, especially for GWAS SNP target genes and lung cancer-related genes (*NF1*, *RNF135*, *FBLN1*, and *FOXO4*).

## Data availability

Sequence data generated in this study have been deposited in the Genome Sequence Archive for Human [65] at the National Genomics Data Center, Beijing Institute of Genomics, Chinese Academy of Sciences / China National Center for Bioinformation (GSA-Human: HRA000295 with BioProject: PRJCA003299), and are publicly accessible at https://ngdc.cncb.ac.cn/gsa-human/.

## Competing interests

The authors have declared no competing interests.

## CRediT authorship contribution statement

**Yan Zhang:** Conceptualization, Investigation, Validation, Writing – review & editing. **Jingwen Zhang:** Formal analysis, Data curation, Visualization, Writing – review & editing. **Wei Zhang:** Investigation. **Mohan Wang:** Investigation. **Shuangqi Wang:** Data curation. **Yao Xu:** Investigation. **Lun Zhao:** Investigation. **Xingwang Li:** Conceptualization, Writing – review & editing. **Guoliang Li:** Conceptualization, Supervision, Writing – review & editing, Funding acquisition. All authors have read and approved the final manuscript.

## Supplementary material

Supplementary Figure S1 **Reproducibility of ChIA-PET data A.** Scatter plots showing contact matrix correlation between different ChIA-PET replicates in A549 cells. **B.** Comparison of ChIA-PET interaction clusters between two RNAPII replicates and two EZH2 replicates represented by chromosome 1: 43.27–45.64 Mb. Rep, replicate.

Supplementary Figure S2 **Hierarchical 3D genome structures analysis using ChIA-PET, Hi-C, and HiChIP data from other publications A.** Span distribution of paired-end reads from HeLa CTCF ChIA-PET data (GSE72816), showing 10 bp and 190 bp periods. **B.** Span distribution of paired-end reads from GM12878 CTCF ChIA-PET data (GSE72816), showing 10 bp and 190 bp periods. **C.** Span distribution of paired-end reads from mESCs SUZ12 ChIA-PET data (GSE120393), showing 10 bp and 190 bp periods. **D.** Span distribution of valid pairs from A549 Hi-C data (ENCODE: ENCSR662QKG). There is no 10 bp or 190 bp period. **E.** Span distribution of valid pairs from IMR-90 Hi-C data (ENCODE: ENCSR852KQC). There is no 10 bp or 190 bp period. **F.** Span distribution of valid pairs from HeLa CTCF HiChIP data (GSE108869). There is no 10 bp or 190 bp period. **G.** Span distribution of valid pairs from GM12878 CTCF HiChIP data (GSE115524). There is no 10 bp or 190 bp period. In all panels, X-axis represents spans of valid pairs in bp. mESCs, mouse embryonic stem cells; HiChIP, *in situ* Hi-C followed by chromatin immunoprecipitation.

Supplementary Figure S3 **Comparison of factor-specific chromatin interactions A.** Distribution of PCCs for gene pairs with promoter–promoter interactions, randomly rewired gene pairs, and randomly selected gene pairs from control regions with the same genomic span. P–P represents gene pairs with promoter–promoter interactions. **B.** Proportional distribution of four classes of enhancers and repressors observed in A549 cells based on locations relative to gene coding regions. “Intra-genic proximal” enhancers are located inside a gene body and interact with nearby promoters; “Extra-genic proximal” enhancers are located outside a gene body and interact with nearby promoters; “Intra-genic distal” enhancers are located inside a gene body, bypass nearby genes, and interact with distant gene promoters over long distances; “Extra-genic distal” enhancers are located outside all gene bodies bypass nearby genes, and interact with distant gene promoters over long distances. **C.** Comparison of expression levels of genes with different types of promoter–enhancer or promoter–repressor interactions. *P* value was determined using one-sided Mann-Whitney U test. **D.** Expression levels of genes with promoters with or without EZH2 or H3K27me3 binding or loop anchor binding. *P* value was determined using Mann-Whitney U test, ***, *P* < 0.001. “+”, genes are located in peak or loop anchor regions; “−”, genes are not located in peak or loop anchor regions. **E.** Examples of EIDs in heatmap. **F.** Densities of active genes (FPKM > 1) per 1 Mb in EIDs, HIDs, and RIDs. **G.** Percentage of EIDs, HIDs, and RIDs located in A or B compartments.

Supplementary Figure S4 **Lung cancer-related genes and SNPs involved in chromatin interactions A.** PCCs for *ERBB2* versus interacting genes in normal samples from TCGA LUAD dataset. Genes are the same as those in Figure 4C. Coexpression pattern was much weaker in normal samples. **B.** Interaction loops on survival-related gene *DKK1* in A549 and BEAS-2B cells. Upper track in each box shows interaction loops and peak signals. EZH2 loops were not observed in A549 cell line. Based on RNA-seq signals, *DKK1* showed higher expression in A549 than in BEAS-2B cells. **C.** Comparison of gene expression in tumor and normal samples from TCGA LUAD dataset. Genes interacting with lung cancer risk-related SNPs are marked with red dots. Genes not differentially expressed are marked with gray dots. Up- and down-regulated genes in tumor samples are marked with yellow and blue dots, respectively.

Supplementary Figure S5 **Genes with different interactions and expression levels in A549 and BEAS-2B cell lines A.** Venn diagrams of genes with differential RNAPII interactions and differentially expressed in A549 and BEAS-2B cell lines. **B.** Venn diagrams of genes with differential EZH2 interactions and differentially expressed in A549 and BEAS-2B cell lines. Red circles represent genes with specific chromatin interactions in A549 cells. Green circles represent genes with specific chromatin interactions in BEAS-2B cells. Purple circles represent genes with significantly differential expression levels (adjusted *P* value < 0.01, |log2 fold change| > 1) in both cell lines. **C.** Differential RNAPII interactions associated with *HOXB* cluster genes in A549 and BEAS-2B cell lines. Based on RNA-seq tracks, these genes showed higher expression in A549 than in BEAS-2B cells. **D.** GO analysis terms of specific RNAPII interactions and differentially expressed genes in BEAS-2B cells. GO, Gene Ontology.

Supplementary Figure S6 **Expression profiles of cancer-related gene *FBLN1* in TCGA database A.** Differential EZH2 interactions associated with *FBLN1* in A549 and BEAS-2B cell lines. Based on RNA-seq tracks, *FBLN1* showed lower expression in A549 than in BEAS-2B cells. **B.** Boxplots of distribution of *FBLN1* mRNA expression levels in a large set of LUAD tumor tissues and normal tissues from TCGA database. *P* value was determined using Mann-Whitney U test. ***, *P* < 0.001.

Supplementary Table S1 **Summary information of ChIA-PET libraries**

Supplementary Table S2 **Lung cancer genes and those involved in chromatin interactions**

Supplementary Table S3 **Top 100 LUAD survival risk genes and those involved in chromatin interactions**

Supplementary Table S4 **Lung cancer related GWAS SNPs and those involved in chromatin interactions**

Supplementary Table S5 **Differentially expressed genes between A549 and BEAS-2B cell lines**
