# Supplementary figures and images for: Mapping Multi-factor-mediated Chromatin Interactions to Assess Dysregulation of Lung Cancer-related Genes
Source: Genomics Proteomics Bioinformatics. 2023 Jan 23;21(3):573–88. doi: 10.1016/j.gpb.2023.01.004 (PMC10787015; doi:10.1016/j.gpb.2023.01.004)

**A**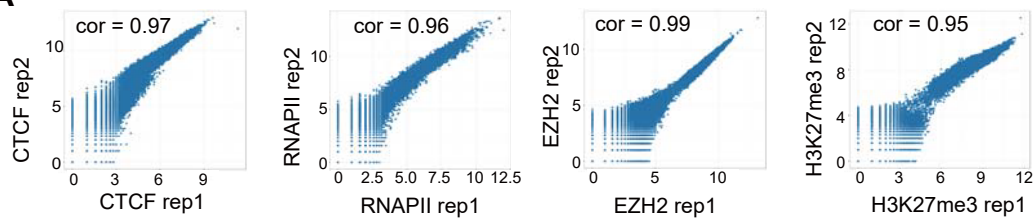**B**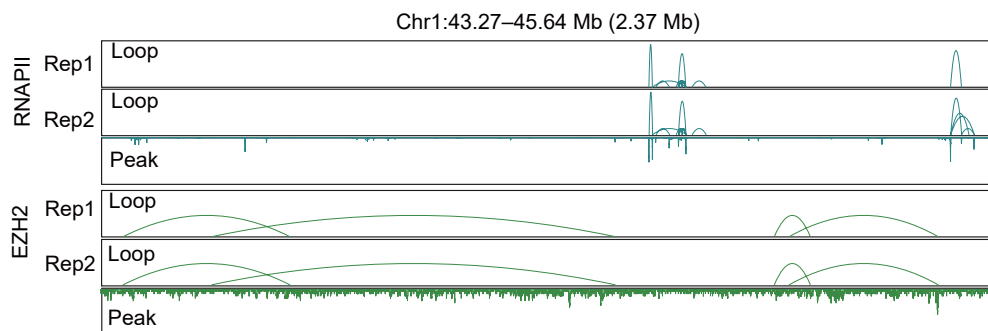

Supplement: Supplementary Figure S1 — Reproducibility of ChIA-PET data A. Scatter plots showing contact matrix correlation between different ChIA-PET replicates in A549 cells. B. Comparison of ChIA-PET interaction clusters between two RNAPII replicates and two EZH2 replicates represented by chromosome 1: 43.27–45.64 Mb. Rep, replicate. [file mmc1.pdf]

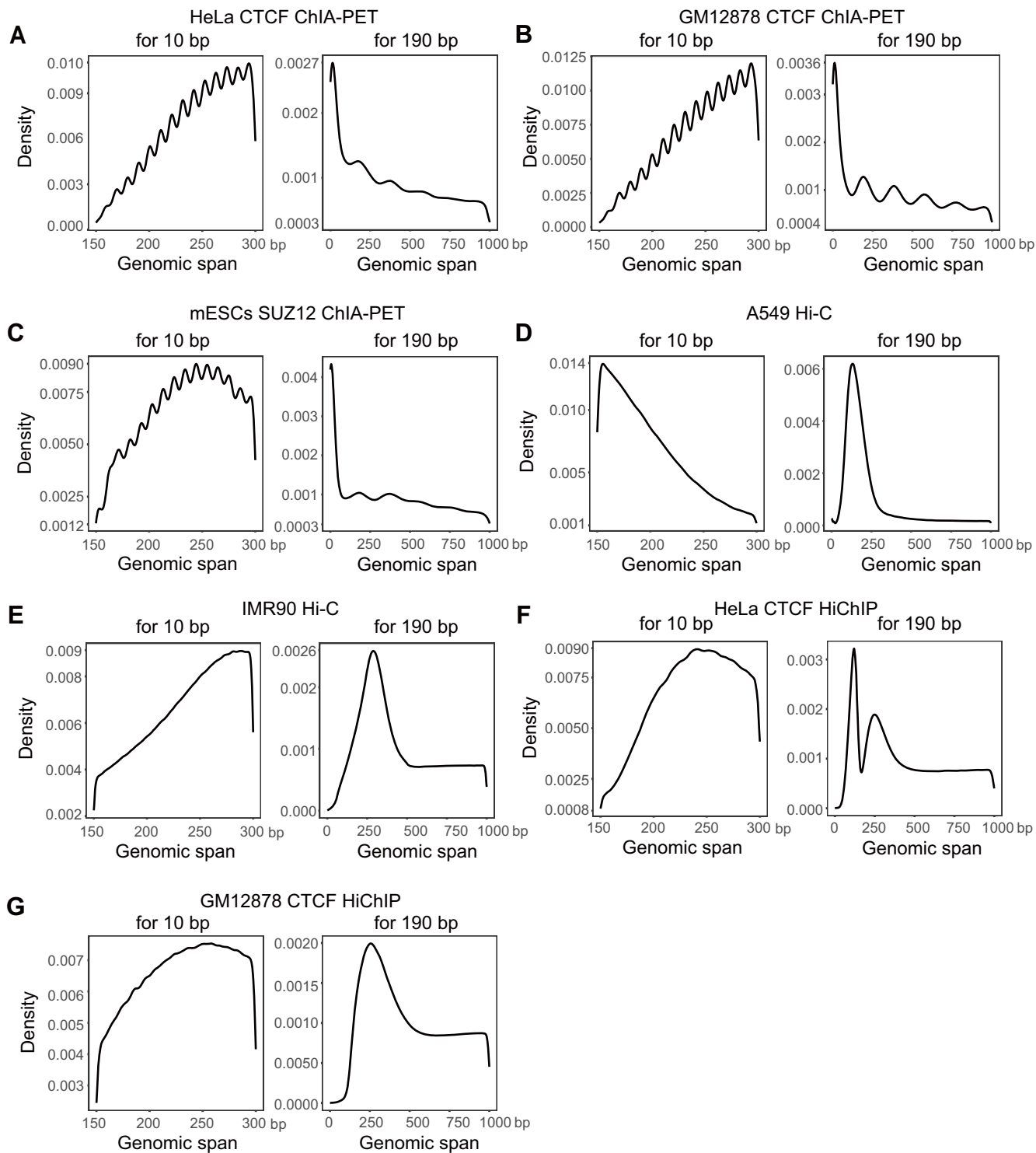

Supplement: Supplementary Figure S2 — Hierarchical 3D genome structures analysis using ChIA-PET, Hi-C, and HiChIP data from other publications A. Span distribution of paired-end reads from HeLa CTCF ChIA-PET data (GSE72816), showing 10 bp and 190 bp periods. B. Span distribution of paired-end reads from GM12878 CTCF ChIA-PET data (GSE72816), showing 10 bp and 190 bp periods. C. Span distribution of paired-end reads from mESCs SUZ12 ChIA-PET data (GSE120393), showing 10 bp and 190 bp periods. D. Span distribution of valid pairs from A549 Hi-C data (ENCODE: ENCSR662QKG). There is no 10 bp or 190 bp period. E. Span distribution of valid pairs from IMR-90 Hi-C data (ENCODE: ENCSR852KQC). There is no 10 bp or 190 bp period. F. Span distribution of valid pairs from HeLa CTCF HiChIP data (GSE108869). There is no 10 bp or 190 bp period. G. Span distribution of valid pairs from GM12878 CTCF HiChIP data (GSE115524). There is no 10 bp or 190 bp period. In all panels, X-axis represents spans of valid pairs in bp. mESCs, mouse embryonic stem cells; HiChIP, in situ Hi-C followed by chromatin immunoprecipitation. [file mmc2.pdf]

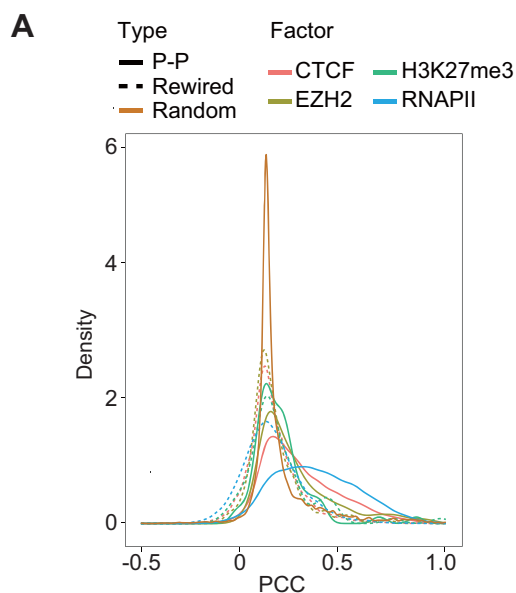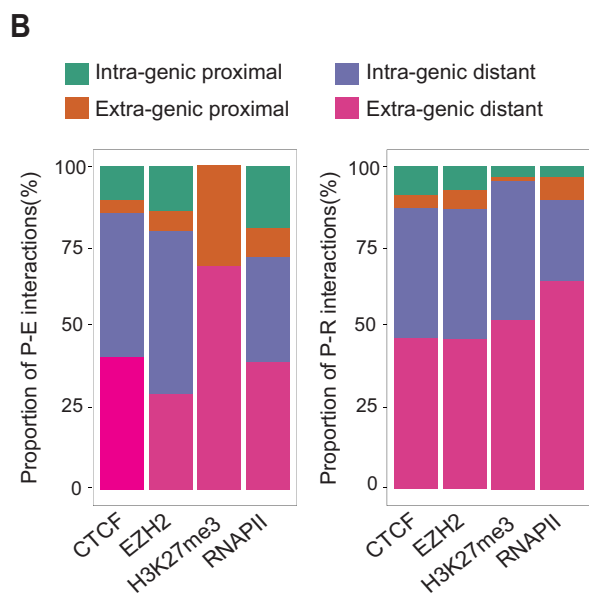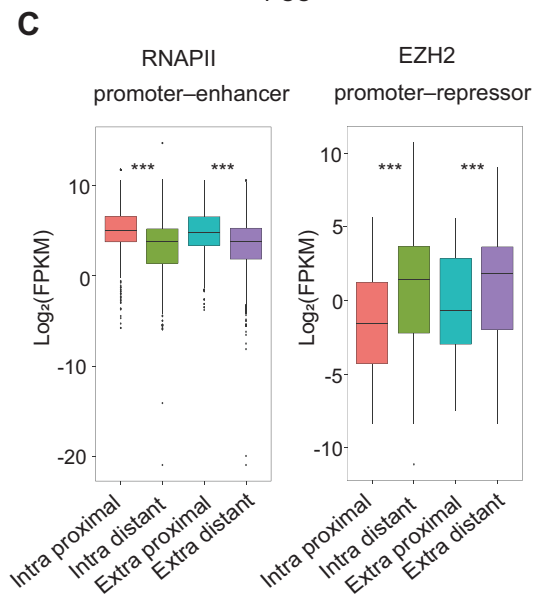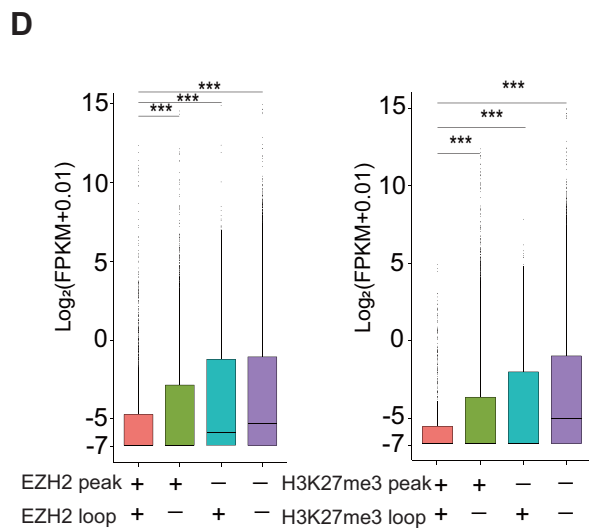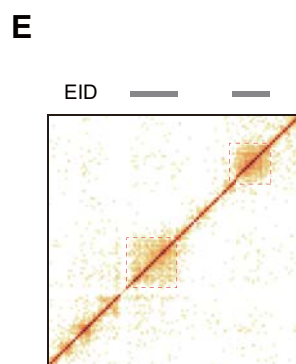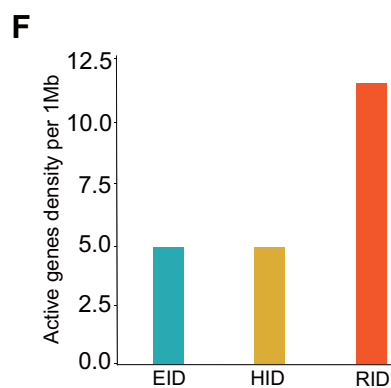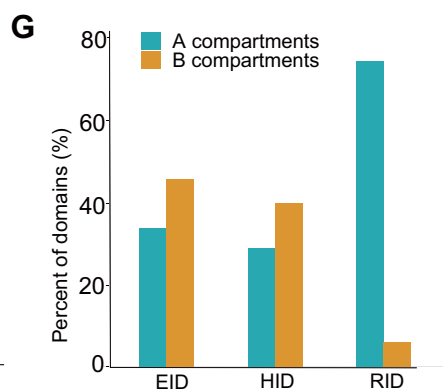

Supplement: Supplementary Figure S3 — Comparison of factor-specific chromatin interactions A. Distribution of PCCs for gene pairs with promoter–promoter interactions, randomly rewired gene pairs, and randomly selected gene pairs from control regions with the same genomic span. P–P represents gene pairs with promoter–promoter interactions. B. Proportional distribution of four classes of enhancers and repressors observed in A549 cells based on locations relative to gene coding regions. “Intra-genic proximal” enhancers are located inside a gene body and interact with nearby promoters; “Extra-genic proximal” enhancers are located outside a gene body and interact with nearby promoters; “Intra-genic distal” enhancers are located inside a gene body, bypass nearby genes, and interact with distant gene promoters over long distances; “Extra-genic distal” enhancers are located outside all gene bodies bypass nearby genes, and interact with distant gene promoters over long distances. C. Comparison of expression levels of genes with different types of promoter–enhancer or promoter–repressor interactions. P value was determined using one-sided Mann-Whitney U test. D. Expression levels of genes with promoters with or without EZH2 or H3K27me3 binding or loop anchor binding. P value was determined using Mann-Whitney U test, ***, P < 0.001. “+”, genes are located in peak or loop anchor regions; “−”, genes are not located in peak or loop anchor regions. E. Examples of EIDs in heatmap. F. Densities of active genes (FPKM > 1) per 1 Mb in EIDs, HIDs, and RIDs. G. Percentage of EIDs, HIDs, and RIDs located in A or B compartments. [file mmc3.pdf]

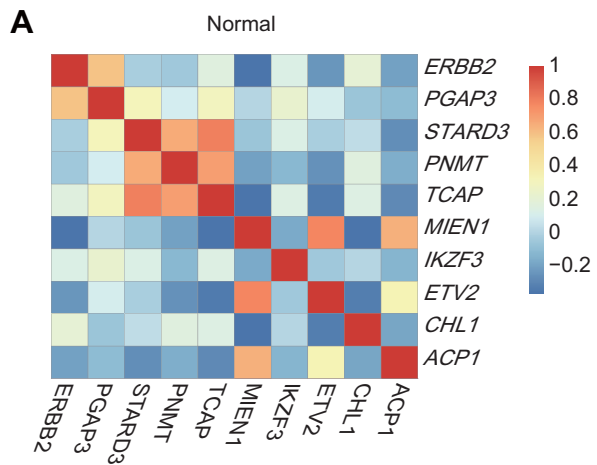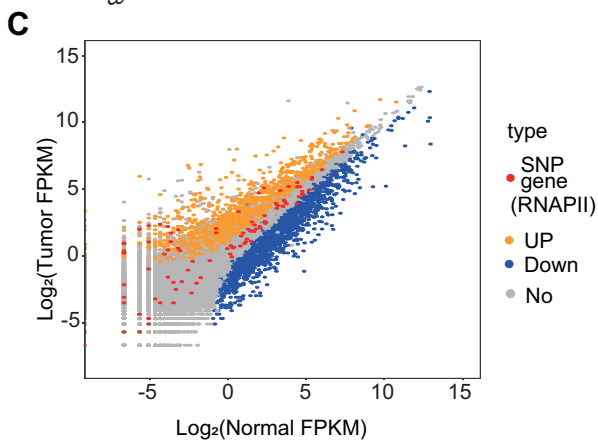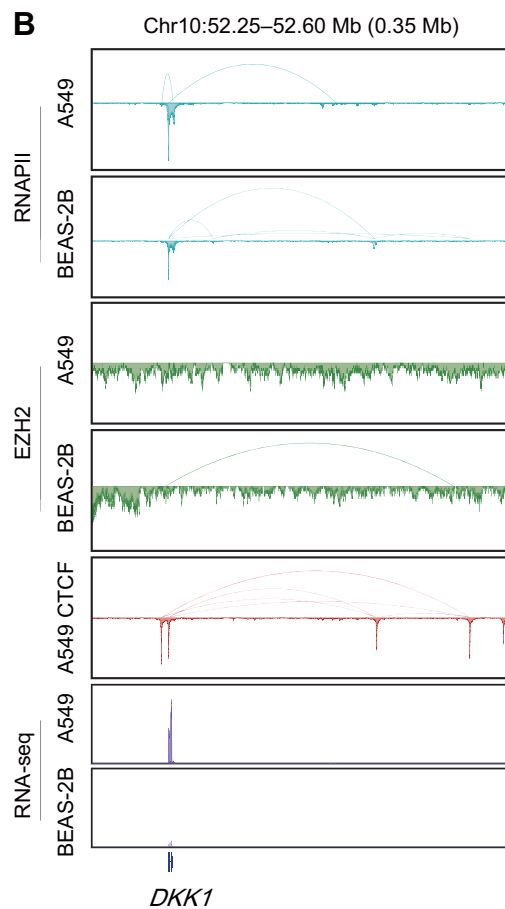

Supplement: Supplementary Figure S4 — Lung cancer-related genes and SNPs involved in chromatin interactions A. PCCs for ERBB2 versus interacting genes in normal samples from TCGA LUAD dataset. Genes are the same as those in Figure 4C. Coexpression pattern was much weaker in normal samples. B. Interaction loops on survival-related gene DKK1 in A549 and BEAS-2B cells. Upper track in each box shows interaction loops and peak signals. EZH2 loops were not observed in A549 cell line. Based on RNA-seq signals, DKK1 showed higher expression in A549 than in BEAS-2B cells. C. Comparison of gene expression in tumor and normal samples from TCGA LUAD dataset. Genes interacting with lung cancer risk-related SNPs are marked with red dots. Genes not differentially expressed are marked with gray dots. Up- and down-regulated genes in tumor samples are marked with yellow and blue dots, respectively. [file mmc4.pdf]

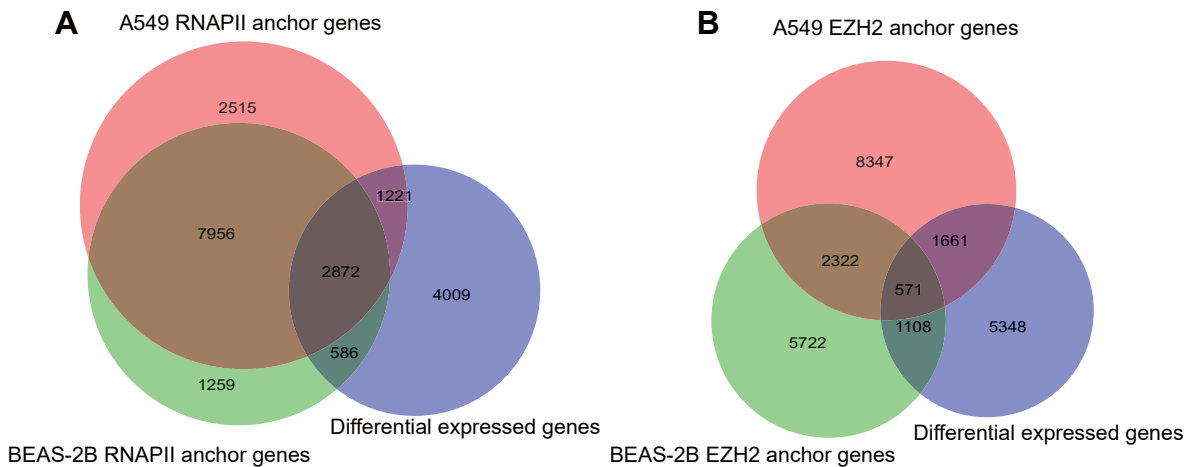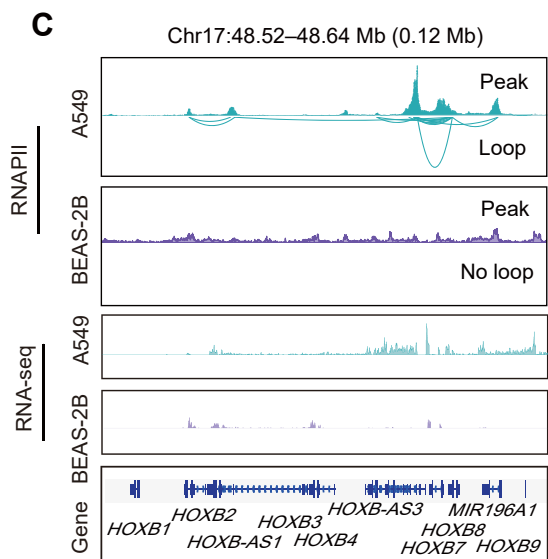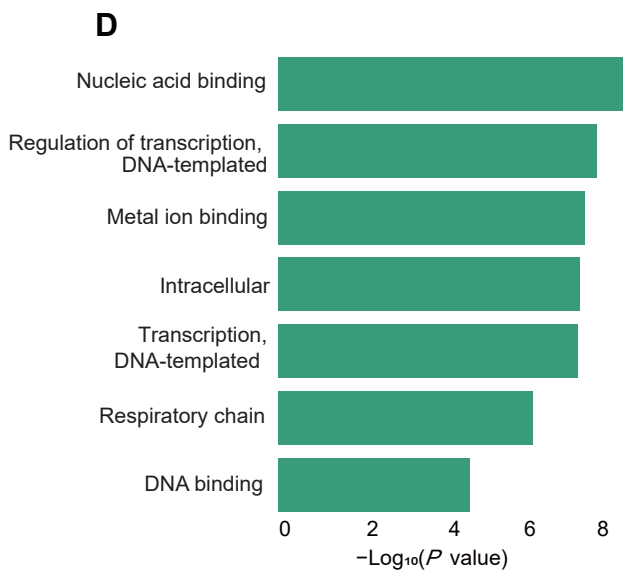

Supplement: Supplementary Figure S5 — Genes with different interactions and expression levels in A549 and BEAS-2B cell lines A. Venn diagrams of genes with differential RNAPII interactions and differentially expressed in A549 and BEAS-2B cell lines. B. Venn diagrams of genes with differential EZH2 interactions and differentially expressed in A549 and BEAS-2B cell lines. Red circles represent genes with specific chromatin interactions in A549 cells. Green circles represent genes with specific chromatin interactions in BEAS-2B cells. Purple circles represent genes with significantly differential expression levels (adjusted P value < 0.01, |log2 fold change| > 1) in both cell lines. C. Differential RNAPII interactions associated with HOXB cluster genes in A549 and BEAS-2B cell lines. Based on RNA-seq tracks, these genes showed higher expression in A549 than in BEAS-2B cells. D. GO analysis terms of specific RNAPII interactions and differentially expressed genes in BEAS-2B cells. GO, Gene Ontology. [file mmc5.pdf]

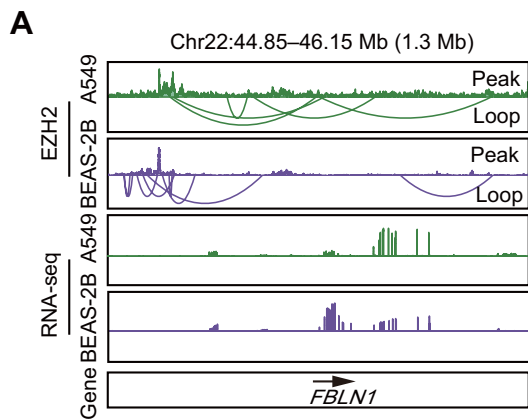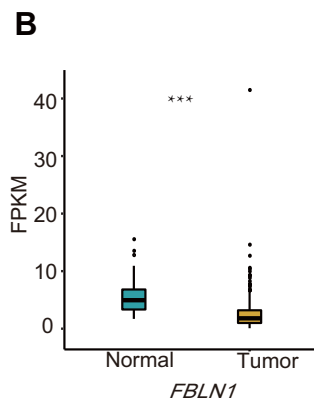

Supplement: Supplementary Figure S6 — Expression profiles of cancer-related gene FBLN1 in TCGA database A. Differential EZH2 interactions associated with FBLN1 in A549 and BEAS-2B cell lines. Based on RNA-seq tracks, FBLN1 showed lower expression in A549 than in BEAS-2B cells. B. Boxplots of distribution of FBLN1 mRNA expression levels in a large set of LUAD tumor tissues and normal tissues from TCGA database. P value was determined using Mann-Whitney U test. ***, P < 0.001. [file mmc6.pdf]
